# Supplementary material for: Content-rich biological network constructed by mining PubMed abstracts
Source: BMC Bioinformatics. 2004 Oct 8;5:147. doi: 10.1186/1471-2105-5-147 (PMC528731; doi:10.1186/1471-2105-5-147)
Supplement: Additional File 2 — The original results of the above study (non-essential files are deleted to keep the file size under the limit set by BMC bioinformatics). [file 1471-2105-5-147-S2.bz2 › chilibotAdditionalFile2/dip50/219ID7617032E728/html/IFNGR1_IFNG.html]

 


 **IFNGR1** and **IFNG** 
  
Found 72 abstracts in PubMed, retrieved 50.  
 

 What does Google say? 
 PDF only 
| .edu only 

---

**Interactive relationship** (e.g. stimulation, inhibition, etc)

**Stimulatory relationship**- IFN gamma  [ **IFNG** ]  receptor chain 1 IFNGR 1  [ **IFNGR1** ]  tyrosine phosphorylation is markedly enhanced in cells treated with TNF alpha IFN gamma  [ **IFNG** ]  without alteration in receptor levels.  Ref: 10454343 J Interferon Cytokine Res, 1999
- Isolation of lipid microdomains from Jurkat cells showed that both IFNGR 1  [ **IFNGR1** ]  and IFNGR  [ **IFNGR1** ]  2 were associated with lipid microdomains only after stimulation with IFN gamma  [ **IFNG** ] , suggesting that the IFNGR  [ **IFNGR1** ]  subunits are recruited to lipid microdomains by IFN gamma  [ **IFNG** ]  binding in lymphocytes Jurkat in contrast to their constitutive presence in epithelial WISH cells.  Ref: 12165521 J Immunol, 2002
**Neutral relationship**- Thus, membrane lipid microdomains play an important role in IFN gamma  [ **IFNG** ]  initiated endocytic events involving IFNGR 1  [ **IFNGR1** ] , and the nuclear translocation of IFN gamma  [ **IFNG** ] , IFNGR 1  [ **IFNGR1** ] , and STAT1alpha.  Ref: 12165521 J Immunol, 2002
- However, treatment of cells with IFN gamma  [ **IFNG** ]  resulted in rapid migration of IFNGR 1  [ **IFNGR1** ] , but not IFNGR  [ **IFNGR1** ]  2, from these microdomains.  Ref: 12165521 J Immunol, 2002
- A multipoint LOD score of 3.1 was obtained at  **IFNGR1** , the gene that encodes chain 1 of the interferon gamma  [ **IFNG** ]  IFN gamma  [ **IFNG** ]  receptor.  Ref: 12516030 Am J Hum Genet, 2003
- IFN gamma  [ **IFNG** ]  contains a nuclear localization sequence that may play a role in the nuclear transport of activated STAT1alpha via a complex of IFN gamma  [ **IFNG** ]  IFN gamma  [ **IFNG** ]  receptor IFNGR 1  [ **IFNGR1** ]  STAT1alpha with the nuclear importer nucleoprotein interactor 1.  Ref: 12165521 J Immunol, 2002
- Mutations in  **IFNGR1**  and IFNGR2, encoding the two IFN gamma  [ **IFNG** ]  receptor chains, and mutations in STAT1, encoding an essential signaling component, result in impaired cellular responses to IFN gamma  [ **IFNG** ] .  Ref: 11213797 Immunol Rev, 2000
- These data suggest that reduced IFNGR  [ **IFNGR1** ]  expression may be responsible for immune cell hyporesponsiveness to IFN gamma  [ **IFNG** ] , which may, in part, explain some of the immunomodulatory and anti inflammatory effects associated with the consumption of diets high in n 3 PUFA.  Ref: 10048767 J Interferon Cytokine Res, 1999

**Non-interactive relationship** (e.g. studied together, co-existance, homology, etc.)

- The purpose of this study was to investigate whether the amino acid polymorphism Val14Met found within the IFN gamma  [ **IFNG** ]  receptor gene  **IFNGR1**  plays a prominent role in susceptibility to SLE.  Ref: 10079289 Immunogenetics, 1999
- These lately discovered genes, relevant to immune disorders of mononuclear phagocytes and neutrophils, include defects in the interferon gamma  [ **IFNG** ]   **IFNg**  interleukin 12 IL 12 pathway, such as  **IFNg**  receptor IFNgR  [ **IFNGR1** ]  defects, IL 12 defect, IL 12 receptor IL 12R defect, and signal transducer and activator of transcription 1 STAT 1 defect.  Ref: 12165204 Curr Allergy Asthma Rep, 2002
- Interleukin 12 receptor beta 1 IL12RB1, interleukin 12 receptor beta 2 IL12RB2, and interferon gamma  [ **IFNG** ]  receptor 1  [ **IFNGR1** ]   **IFNGR1**  perform important roles in the host defense against intracellular pathogens such as Mycobacteria.  Ref: 12743658 Immunogenetics< MedlineTA>Immunogenetics, 2002
- Identification of a phorbol ester responsive element in the interferon gamma  [ **IFNG** ]  receptor 1  [ **IFNGR1** ]  chain gene.  Ref: 11477089 J Biol Chem, 2001
- Genetic basis of patients with bacille Calmette Guérin osteomyelitis in Japan identification of dominant partial interferon gamma  [ **IFNG** ]  receptor 1  [ **IFNGR1** ]  deficiency as a predominant type.  Ref: 11865431 J Infect Dis, 2002
- Interferon gamma  [ **IFNG** ]  receptor 1  [ **IFNGR1** ]  IFNgammaR1 deficiency is a rare inherited immunodeficiency.  Ref: 12805054 Blood< MedlineTA>Blood, 2002
- D6S471, a microsatellite marker on chromosome 6 near interferon gamma  [ **IFNG** ]  receptor 1  [ **IFNGR1** ] , was amplified with polymerase chain reaction, and genotypes were determined.  Ref: 10923008 Surgery, 2000
- The genes for the interferon gamma  [ **IFNG** ]  receptor 1  [ **IFNGR1** ]  and the natural resistance associated macrophage protein 1 NRAMP1 control the immune response to intracellular microbial pathogens.  Ref: 10207725 Int J Colorectal Dis, 1999
- Analysis of antibody A6 binding to the extracellular interferon gamma  [ **IFNG** ]  receptor alpha chain  [ **IFNGR1** ]  by alanine scanning mutagenesis and random mutagenesis with phage display.  Ref: 11123892 Biochemistry, 2000
- Among the 12 down regulated genes, interferon gamma  [ **IFNG** ]  receptor 1  [ **IFNGR1** ]  was most notable x1 27.3.  Ref: 12823984 Exp Eye Res, 2003
- Radiation of the esophagus of C3H HeNsd mice with 35 or 37 Gy of 6 MV X rays induces significantly increased RNA transcription for interleukin 1 Il1, tumor necrosis factor alpha Tnf, interferon gamma  [ **IFNG** ]  inducing factor Ifngr  [ **IFNGR1** ] , and interferon gamma  [ **IFNG** ]   **Ifng** .  Ref: 11121210 Radiat Res, 2001
- Different types of mutations in four genes  **IFNGR1** , IFNGR2, IL12B, IL12RB1 have revealed both allelic and nonallelic heterogeneity and result in eight different disorders whose common pathogenic pathway is impaired interferon gamma  [ **IFNG** ]  IFNgamma mediated immunity.  Ref: 11420412 Pediatr Res, 2001
- Filipin pretreatment, which specifically inhibits endocytosis from caveolae like microdomains, inhibited the nuclear translocation of IFN gamma  [ **IFNG** ]  and IFNGR 1  [ **IFNGR1** ]  as well as the tyrosine phosphorylation and nuclear translocation of STAT1alpha, but did not affect the binding of IFN gamma  [ **IFNG** ]  to these cells.  Ref: 12165521 J Immunol, 2002
- The combination of polymorphisms within interferon gamma  [ **IFNG** ]  receptor 1  [ **IFNGR1** ]  and receptor 2 associated with the risk of systemic lupus erythematosus.  Ref: 10403400 FEBS Lett, 1999
- Persistent Mycobacterium avium infection following non myeloablative allogeneic peripheral blood stem cell transplantation for Interferon gamma  [ **IFNG** ]  receptor 1  [ **IFNGR1** ]  deficiency.  Ref: 12805054 Blood< MedlineTA>Blood, 1999
- Recently, recessive mutations in the interferon gamma  [ **IFNG** ]  receptor ligand binding chain  **IFNgR1** , interferon gamma  [ **IFNG** ]  receptor signalling chain IFNgR2, interleukin 12 p40 subunit IL 12p40, and interleukin 12 receptor beta 1 chain IL 12Rb1 genes have been identified in a number of patients with disseminated BCG or NTM infection.  Ref: 11261247 Bull Acad Natl Med, 2000
- Antileishmanial drugs cause up regulation of interferon gamma  [ **IFNG** ]  receptor 1  [ **IFNGR1** ] , not only in the monocytes of visceral leishmaniasis cases but also in cultured THP1 cells.  Ref: 12803856 Ann Trop Med Parasitol, 2003
- Both surface and intracellular staining of all cell lines with antibodies against  **IFNgR1**  and IFNgR2 failed to detect any increase in  **IFNg**  receptor expression following incubation with PMA.  Ref: 12787470 Cancer Cell Int< MedlineTA>Cancer Cell Int, 2003
- Cross phosphorylation of  **IFNGR1**  is not required for the IFN gamma  [ **IFNG** ]  like response through the chimeric receptors, nor does it mediate an IFN gamma  [ **IFNG** ]  like response to IL 6 or OSM.  Ref: 11574475 EMBO J, 2001
- Linkage analysis on Con A blasts from BALB c x CByB6F1 N2 backcross progeny for 1 relative level of competitive inhibition of BALB c NK lysis of syngeneic Con A blasts and 2 genotypes at polymorphic microsatellite markers distributed throughout the mouse genome mapped the Nktar gene locus to a 5 cM region of chromosome 10 containing the interferon gamma  [ **IFNG** ]  receptor Ifngr  [ **IFNGR1** ]  gene locus.  Ref: 10753500 Cell Immunol, 2000
- Complete interferon gamma  [ **IFNG** ]  receptor 1  [ **IFNGR1** ]  IFNgammaR1 deficiency is a primary immunodeficiency disease characterized by high susceptibility to recurrent, severe mycobacterial and other intracellular infections.  Ref: 12393576 Blood, 2002
- Furthermore, the numbers of SEB induced CD80 monocytes were partially decreased by anti CD119  [ **IFNGR1** ]  IFN gamma  [ **IFNG** ]  receptor MAb and by anti CD95L NOK2 MAb.  Ref: 11349026 Infect Immun, 2001
- Reporter gene analyses of 5 deletion constructs of the IFN gamma  [ **IFNG** ]  receptor 1 gene  **IFNGR1**  promoter indicated that the critical region for control of transcription and the TPA responsive element TRE were present in the 128 to 109 base pair bp region.  Ref: 11477089 J Biol Chem, 2001
- We hypothesized that the n 3 PUFA induced hyporesponsiveness to IFN gamma  [ **IFNG** ]  is mediated, in part, by a reduction in the number of IFN gamma  [ **IFNG** ]  receptors IFNGR  [ **IFNGR1** ]  expressed on the surface of these cells.  Ref: 10048767 J Interferon Cytokine Res, 1999
- Using an intronic CA n polymorphic microsatellite marker within the IFN gamma  [ **IFNG** ]  receptor 1  **IFNGR1**  gene, we have compared the allelic frequencies of this marker in hospitalized tuberculosis patients n = 120 with that of controls n = 87 from Rijeka, Croatia.  Ref: 12753505 Scand J Immunol, 2003
- Patients with a dominant small deletion 818del4, hotspot in the interferon gamma  [ **IFNG** ]  receptor 1  [ **IFNGR1** ]   **IFNGR1**  gene 6q23 q24 and increased susceptibility to mycobacterial infections have been recently reported.  Ref: 11781064 Clin Immunol, 2002
- METHODS We investigated the association of  **IFNG**  CA repeat polymorphism within the first intron, IRF1 GT repeat polymorphism within the intron 7,  **IFNGR1**  Val 14 Met, and IFNGR2 Gln 64 Arg gene polymorphisms with atopic asthma in the Japanese child population.  Ref: 11240951 J Allergy Clin Immunol, 2001
- BACKGROUND This study hypothesizes that predicted polymorphism of the interferon gamma  [ **IFNG** ]  receptor 1  [ **IFNGR1** ]  gene may play an important role in infection after trauma as supported by microsatellite analysis.  Ref: 10923008 Surgery, 2000
- mutations in interleukin 12 receptor beta 1 and interferon gamma  [ **IFNG** ]  receptor 1  [ **IFNGR1** ]  lead to nontuberculous mycobacterial infections and salmonellosis .  Ref: 11020837 Ned Tijdschr Geneeskd, 2000
- Inflammatory bowel disease and the genes for the natural resistance associated macrophage protein 1 and the interferon gamma  [ **IFNG** ]  receptor 1  [ **IFNGR1** ] .  Ref: 10207725 Int J Colorectal Dis, 1999
- Whereas TGF beta R CD105, MCSFR CD115, SCFR CD117, IL 4R IL 13R CD124 and IL 6R CD126 were not expressed, the cell lines were mostly positive for IFN gamma  [ **IFNG** ]  R CD119  [ **IFNGR1** ] , IL 7R CD127 and FLT 3R CD135.  Ref: 10363160 Hum Cell, 1998
- We identified a novel heterozygous single nucleotide substitution 1400 T right curved arrow C Leu 467 Pro in the seventh exon of the interferon gamma  [ **IFNG** ]  receptor 1  [ **IFNGR1** ]   **IFNGR1**  gene.  Ref: 12851715 Int J Mol Med, 2003
- This correlated with reduced expression of IFN gamma  [ **IFNG** ]  R CD119  [ **IFNGR1** ]  by mature DC.  Ref: 10092774 J Immunol, 1999
- Since interferon gamma  [ **IFNG** ]  is the main cytokine responsible for defence against leishmanial parasites, it was thought possible that effective antileishmanial drugs may up regulate  **IFNGR1** .  Ref: 12803856 Ann Trop Med Parasitol, 2003
- these patients had either deficiencies in the interferon gamma  [ **IFNG** ]  receptor 1  [ **IFNGR1** ]  or idiopathic immune defects.  Ref: 10880373 Am J Pathol, 2000
- A novel single nucleotide substitution, Leu 467 Pro, in the interferon gamma  [ **IFNG** ]  receptor 1  [ **IFNGR1** ]  gene associated with allergic diseases.  Ref: 12851715 Int J Mol Med, 2003
- In contrast, treatments that block clathrin dependent endocytosis did not inhibit either activation or nuclear translocation of STAT1alpha or the nuclear translocation of IFN gamma  [ **IFNG** ]  or IFNGR 1  [ **IFNGR1** ] .  Ref: 12165521 J Immunol, 2002
- Mouse strains with null mutations in the gamma interferon gene  **Ifng**  or the gamma interferon receptor gene Ifngr  [ **IFNGR1** ]  have been engineered.  Ref: 10233988 J Virol, 1999
- In contrast, IFN gamma  [ **IFNG** ]  receptor IFNGR  [ **IFNGR1** ]  expression was increased in the presence of anti CD2 mAb.  Ref: 12744772 J Interferon Cytokine Res, 2003
- 561del4 defines a novel small deletion hotspot in the interferon gamma  [ **IFNG** ]  receptor 1  [ **IFNGR1** ]  chain.  Ref: 11781064 Clin Immunol, 2002
- Also, the mutations in the interferon gamma  [ **IFNG** ]  receptor 1  [ **IFNGR1** ]  were not found in the 186 IBD patients.  Ref: 10207725 Int J Colorectal Dis, 1999
- By Western blotting the plasma membrane raft fractions obtained using either non detergent or detergent based methods contained significant amounts of STAT1 and STAT3 up to approximately 10% of the total cytoplasmic amount as well as the integral raft proteins caveolin 1 and flotillin 1, the IL 6 receptor signal transducing chain gp130, the interferon gamma  [ **IFNG** ]  receptor alpha chain  [ **IFNGR1** ]  IFN gammaRalpha, and the chaperone glucose regulated protein 58 GRP58 ER 60 ERp57.  Ref: 11815625 J Biol Chem, 2002
- Microsatellite marker of interferon gamma  [ **IFNG** ]  receptor 1  [ **IFNGR1** ]  gene correlates with infection following major trauma.  Ref: 10923008 Surgery, 2000
- This study explored the relationship between polymorphisms in the promoter region of the gene encoding IFN gamma  [ **IFNG** ]  receptor 1  **IFNGR1**  and susceptibility to malaria in African children.  Ref: 12023780 J Infect Dis, 2002
- However, direct comparisons between  **Ifng**  GKO and Ifngr  [ **IFNGR1** ]  RGKO mice have been problematic because previously available strains of these mice have had different genetic backgroundsi.e., C57BL 6 and BALB c for GKO mice and 129 Sv Ev for RGKO mice.  Ref: 10233988 J Virol, 1999
- Because the uptake of IFN gamma  [ **IFNG** ]  is a receptor mediated endocytotic process, we examined in this study the fate of both the receptor subunits IFNGR 1  [ **IFNGR1** ]  and IFNGR  [ **IFNGR1** ]  2 of the heterodimeric IFN gamma  [ **IFNG** ]  receptor complex.  Ref: 10888113 J Interferon Cytokine Res, 2000
- Interferon gamma  [ **IFNG** ]  receptor 1  [ **IFNGR1** ]  gene polymorphism in tuberculosis patients from Croatia.  Ref: 12753505 Scand J Immunol, 2003
- Differential nuclear localization of the IFNGR 1  [ **IFNGR1** ]  and IFNGR  [ **IFNGR1** ]  2 subunits of the IFN gamma  [ **IFNG** ]  receptor complex following activation by IFN gamma  [ **IFNG** ] .  Ref: 10888113 J Interferon Cytokine Res, 2000
- We previously showed decreased OPN expression in the granulomas of patients with disseminated mycobacterial infection in the setting of mutation of the IFN gamma  [ **IFNG** ]  receptor 1 IFNGR 1  [ **IFNGR1** ] .  Ref: 12804068 J Interferon Cytokine Res, 2003
- The  **IFNGR1**  signal transducing subunit of the IFN gamma  [ **IFNG** ]  receptor is tyrosine phosphorylated through the chimeric receptors and the endogenous IL 6 and OSM receptors.  Ref: 11574475 EMBO J, 2001
- Expression of IFN gamma  [ **IFNG** ]  and IFNGR 1  [ **IFNGR1** ]  was also identified in isolated microvessels from brains.  Ref: 10805375 J Interferon Cytokine Res, 2000
- Correction of complete interferon gamma  [ **IFNG** ]  receptor 1  [ **IFNGR1** ]  deficiency by bone marrow transplantation.  Ref: 12393576 Blood, 2002
- We studied the allele frequencies of two restriction fragment length polymorphisms in the gene for NRAMP1 and the prevalence of two mutations in the interferon gamma  [ **IFNG** ]  receptor 1  [ **IFNGR1** ]  gene.  Ref: 10207725 Int J Colorectal Dis, 1999
- The cerebrum and cerebellum were collected from young adult 8 10 weeks old and aged 24 26 months old BALB c mice, and the expressions of IFN gamma  [ **IFNG** ]  and IFN gamma  [ **IFNG** ]  receptor 1 IFNGR 1  [ **IFNGR1** ]  mRNA were examined by RT PCR.  Ref: 10805375 J Interferon Cytokine Res, 2000
- Modulation and implications in pathogenesis of interferon gamma  [ **IFNG** ]  receptor 1  [ **IFNGR1** ]  in patients with chronic hepatitis B < ArticleTitle>.  Ref: 12546733 Zhonghua Gan Zang Bing Za Zhi, 2003
- Missense mutations of the interleukin 12 receptor beta 1 IL12RB1 and interferon gamma  [ **IFNG** ]  receptor 1  [ **IFNGR1** ]   **IFNGR1**  genes are not associated with susceptibility to lepromatous leprosy in Korea.  Ref: 12743658 Immunogenetics< MedlineTA>Immunogenetics, 2003
- 16 chromosomes and one copy each of disabled IFN gamma  [ **IFNG** ]  receptor IFNGR  [ **IFNGR1** ]  and IFN alpha beta receptor IFNAR 2 component genes.  Ref: 10714556 J Interferon Cytokine Res, 2000
- Apparently for the first time, the peripheral blood monocytes of individuals with active visceral leishmaniasis VL have been found to show reduced expression of interferon gamma  [ **IFNG** ]  receptor 1  [ **IFNGR1** ]   **IFNGR1** .  Ref: 12803856 Ann Trop Med Parasitol, 2003
